# Deciphering the Role of Maternal Microchimerism in Offspring Autoimmunity: A Narrative Review

**DOI:** 10.3390/medicina60091457

**Published:** 2024-09-05

**Authors:** Alexandra Mpakosi, Rozeta Sokou, Martha Theodoraki, Nicoletta Iacovidou, Vasileios Cholevas, Christiana Kaliouli-Antonopoulou

**Affiliations:** 1 Department of Microbiology, General Hospital of Nikaia “Agios Panteleimon”, 18454 Piraeus, Greece; 2 Neonatal Intensive Care Unit, General Hospital of Nikaia “Agios Panteleimon”, 18454 Piraeus, Greece; anastasiosmmr@yahoo.gr; 3 Neonatal Department, National and Kapodistrian University of Athens, Aretaieio Hospital, 11528 Athens, Greece; niciac58@gmail.com; 4 School of Medicine, University of Bologna, 40126 Bologna, Italy; billcholevas34@gmail.com; 5 Department of Immunology, General Hospital of Nikaia “Agios Panteleimon”, 18454 Piraeus, Greece; c.kalanto@gmail.com

**Keywords:** microchimeric cells, pregnancy, Type 1 diabetes, juvenile idiopathic inflammatory myopathies, systemic lupus erythematosus, Sjögren’s-syndrome

## Abstract

Feto-maternal microchimerism is the bidirectional transfer of cells through the placenta during pregnancy that can affect the health of both the mother and the offspring, even in childhood or adulthood. However, microchimerism seems to have different consequences in the mother, who already has a developed immune system, than in the fetus, which is vulnerable with immature defense mechanisms. Studies have shown that the presence of fetal microchimeric cells in the mother can be associated with reduced fetal growth, pre-eclampsia, miscarriage, premature birth, and the risk of autoimmune disease development in the future. However, some studies report that they may also play a positive role in the healing of maternal tissue, in cancer and cardiovascular disease. There are few studies in the literature regarding the role of maternal microchimeric cells in fetal autoimmunity. Even fewer have examined their association with the potential triggering of autoimmune diseases later in the offspring’s life. The objectives of this review were to elucidate the mechanisms underlying the potential association between maternal cells and autoimmune conditions in offspring. Based on our findings, several hypotheses have been proposed regarding possible mechanisms by which maternal cells may trigger autoimmunity. In Type 1 diabetes, maternal cells have been implicated in either attacking the offspring’s pancreatic β-cells, producing insulin, differentiating into endocrine and exocrine cells, or serving as markers of tissue damage. Additionally, several potential mechanisms have been suggested for the onset of neonatal lupus erythematosus. In this context, maternal cells may induce a graft-versus-host or host-versus-graft reaction in the offspring, function as effectors within tissues, or contribute to tissue healing. These cells have also been found to participate in inflammation and fibrosis processes, as well as differentiate into myocardial cells, potentially triggering an immune response. Moreover, the involvement of maternal microchimeric cells has been supported in conditions such as juvenile idiopathic inflammatory myopathies, Sjögren’s syndrome, systemic sclerosis, biliary atresia, and rheumatoid arthritis. Conversely, no association has been found between maternal cells and celiac disease in offspring. These findings suggest that the role of maternal cells in autoimmunity remains a controversial topic that warrants further investigation.

## 1. Introduction

The compound term “microchimerism” consists of the Greek word “micro”, meaning small, and “chimera”. According to Greek mythology, the chimera was a monster made up of parts of three animals—the head and body of a lion, the head of a goat, and a tail ending in the head of a snake [1].

The term “microchimerism” is often borrowed from biology and genetics and denotes an organism whose cells originate from two or more zygotes [2]. In addition, microchimerism is considered the presence of two genetically different populations of cells in an organ such as bone marrow [1].

In general, microchimerism can be distinguished into natural and artificial. Examples of natural microchimerism are pregnancy, miscarriage, and sexual intercourse. Examples of artificial microchimerism are blood transfusions and bone marrow or solid organ transplantations [1].

During pregnancy, there is a normal exchange of cellular components and other substances across the placenta both from the mother to the fetus and vice versa. Nutrients, water, electrolytes, oxygen, hormones, immunoglobulins, cytokines, metabolites, extracellular vesicles, and microchimeric cells can be transferred from the mother to the fetus, as well as carbon dioxide, products of catabolism, and microchimeric cells from the fetus to the mother [3]. This two-way exchange has different consequences in the mother, who has an already developed immune system, than in the fetus. In particular, the transfer of maternal material to the fetus seems to have a decisive effect on its innate immune system [4].

Previous studies, as early as 1893, reported the presence of fetal cells in the tissues of dead pregnant women, while later research from the 1960s and 1970s demonstrated the transfer of leukocytes from the fetus to the mother’s circulation [4]. As recently shown, fetal microchimerism increases more as gestational age progresses [5]. After delivery, the mother’s immune system is activated and, through apoptosis, removes the fetal cells from the maternal circulation. Nevertheless, some fetal cells may escape the apoptotic process, possibly due to apoptosis-resistant ancestors or defective apoptosis-regulation mechanisms, and remain in the maternal tissues even for many years after delivery [6]. It is not yet clear whether fetal microchimerism can benefit or harm the mother’s health. Previous studies have suggested that fetal cells can play a role in maternal tissue repair, while others have shown a possible association with pregnancy complications, such as reduced fetal development, preeclampsia, miscarriage, and premature labor [7,8,9,10].

Preeclampsia is a serious complication during pregnancy that occurs in two stages. During Stage 1, placental dysfunction begins mainly as a result of either insufficient uteroplacental remodeling or placental compression or aging. Subsequently, excessive syncytiotrophoblast stress has a direct effect on the type and amount of signaling molecules, extracellular vesicles, and trophoblast material released from the placenta into the maternal circulation. It has been suggested that fetal microchimeric cells are also transferred at this stage, contributing to the development of Stage 2 preeclampsia. During Stage 2, the maternal clinical syndrome of preeclampsia manifests, which, due to maternal vascular inflammation and endothelial dysfunction, leads to hypertension and proteinuria and other signs of end-organ dysfunction [9].

Moreover, in the long term, the presence of fetal cells in the mother’s blood and tissues appears to play a positive role in some cases, such as in cancer and cardiovascular disease, and in others, a negative one, such as in the pathogenesis and reactivation of certain autoimmune diseases [3,8,9,10,11].

On the other hand, maternal material transferred to the fetus begins in the second trimester of pregnancy when the placenta matures and can persist after birth with consequences for the health of the offspring during infancy and childhood, as well as adulthood [11]. Maternal microchimerism seems to play a decisive role in the development of the fetus by regulating both its immune system and the rest of the fetal organs. The effect of maternal microchimerism on the nascent fetal immune system and the likelihood of developing any autoimmune disease in the future are largely unclear. However, there is evidence that maternal microchimeric cells may be associated with the development of certain autoimmune diseases because these cells within host tissues could be activated and involved in inflammatory and other processes triggering autoimmunity. Therefore, the objective of this paper is to review and present evidence supporting the association between maternal microchimeric cells and the development of autoimmune diseases in offspring. Understanding the role of maternal microchimerism in autoimmune diseases is crucial, as it offers new insights into disease mechanisms and could lead to innovative therapeutic approaches. By thoroughly analyzing recent findings and addressing existing research gaps, this review aims to provide a comprehensive overview that can guide future investigations, identify potential therapeutic targets, and enhance our understanding of the etiology of autoimmune diseases in relation to maternal–fetal cell interactions.

## 2. Fetal Immune System Development during Pregnancy

The human embryo begins as a one-celled zygote that, after fertilization, will develop into a blastocyst and gastrula. During gastrulation, cells rearrange to form three germ layers, the ectoderm, endoderm, and mesoderm, which then differentiate into distinct types of tissues or organs [12]. Organogenesis covers the period between 3 weeks and 8 weeks of gestation. During this critical period of pregnancy, the developing embryo establishes the foundational structures of all its organs and tissues derived from the ectoderm, mesoderm, and endoderm layers. Specifically, the central and peripheral nervous systems, as well as the skin, sensory organs, hair, and nails, develop from the ectoderm; the skeleton, muscular system, connective tissue, heart, blood vessels, and genitourinary system form from the mesoderm and the gastrointestinal tract, liver, pancreas, and lungs develop from the endoderm. Additionally, organs such as the liver, bone marrow, thymus, spleen, kidneys, intestines, and lungs contribute to the development of the fetus’s immune system. [12]. However, during embryonic development and before the formation of the bone marrow and thymus, hematopoiesis is temporarily covered by the yolk sac mesoderm and the extraembryonic mesenchymal tissue. As early as 3–4 weeks of gestation, granulocyte progenitors and the pluripotent erythroid are observed in the yolk sac. From there, they migrate through the circulation to the liver where, from about 5–6 weeks of pregnancy, hematopoiesis mainly occurs [13]. By the 10th week of pregnancy, the number of nucleated cells increases and the liver grows rapidly. At 11–12 weeks of gestation, the thymus and spleen develop from liver and stem cells, while at 12–19 weeks of gestation, these progenitor cells incorporate up to 68% monocytes, up to 41% neutrophils, and up to 30% eosinophils [12,14]. From this moment, the innate immune system of the fetus is able to be activated by molecular patterns, defend against pathogens, and destroy dead or defective auto-cells. This immune activation and response gradually decline until approximately 29 to 33 weeks of gestation. In addition, the fetal adaptive immune system is able to activate the antigen-specific activation of T-lymphocytes and B-lymphocytes, creating long-term memory cells against pathogens [12,15].

## 3. Self-Tolerance and Autoimmunity

The immune system has the ability to react to foreign antigens and remain inactive against itself. This inactivation is mainly achieved by regulating the activation and activity of T cells [16]. T-cell activation depends on how and in what environment the specific antigen is presented. If it does not happen properly, or if it happens without co-stimulation, T cells may not be activated, or they may be activated but not proliferate and produce interleukin 2 (IL-2) [17,18]. During development, most autoreactive T cells capable of recognizing autoantigens are clonally deleted by central thymic tolerance or negative selection [19,20]. However, despite all tolerance mechanisms some autoreactive T cells produced by the thymus manage to escape central deletion and enter the circulation. In this case, their activity is suppressed by the regulatory T-cell population (Tregs) [21].

Treg cells are a specialized subpopulation of CD4+ T cells that have a T-cell receptor that is activated by self-antigens and are characterized by a high expression of the IL-2 receptor alpha chain (IL-2Rα, CD25), inhibitory cytokines IL-10, TGF-β, and IL-35, and nuclear transcription factor Foxp3, which is important for their development and function [17,19]. Depending on their region of development, there are two main subsets of Treg cells, thymic Treg cells (tTreg), or otherwise natural Treg cells (nTreg) that are developed in the thymus, and induced Treg cells (iTreg) that arise after the differentiation of conventional CD4+ T cells (Tconv) in the periphery after their stimulation with an antigen and in the presence of TGF-β and IL-2 [19].

Maintenance of self-tolerance and prevention of the development of autoimmunity by Tregs is accomplished by mechanisms such as the killing of Tregs target cells, the modification of target cell signaling, the secretion of anti-inflammatory cytokines such as interleukin-10 (IL-10), IL-35, and transforming growth factor β (TGF-β), and the regulation of target cells by exosome-borne microRNAs [22,23,24,25]. On the contrary, when Foxp3 is defective or absent or when Tregs are reduced, the regulation of self-tolerance is disrupted with subsequent development of autoimmune diseases [17]. Treg numerical and functional deficiencies may be due to monogenetic, innate immune errors, or due to multifactorial processes [22].

Contact of the fetus with foreign antigens in utero can also lead, depending on the antigen, to tolerance or immunogenicity. Immunogenicity in the fetus has been associated with prenatal onset of allergy, immunosurveillance against developmental tumorigenesis, and prenatal immunization against pathogens [23].

Furthermore, it has been found that the fetal immune system is capable of producing regulatory T cells (Tregs) that suppress immune responses to self-antigens. In the fetal thymus, Tregs develop from the 12th week of gestation and remain stable throughout pregnancy and infancy. As it has been previously found, fetal Tregs already express FoxP3 and other markers with immunosuppressive properties [22]. Moreover, Mold JE et al. demonstrated that maternal cells that cross the placenta and home to fetal lymph nodes have the ability to provoke the development of CD4+CD25highFoxP3+ Tregs, which suppress the fetal antimaternal immune response [24].

## 4. Maternal Circulation Material That Is Transferred across the Placenta and Stimulates the Fetal Immune System

### 4.1. Immunoglobulins

Maternal IgG is the only antibody class that is transported across the placenta. Maternofetal IgG transfer begins at 13 weeks gestation and occurs throughout pregnancy, with the majority transferred in the third trimester [26,27]. It has been shown to be incorporated by endocytosis into syncytiotrophoblast contact regions and binds to the neonatal receptor for the crystallizable (Fc) region of the IgG fragment (FcRn) on endosomes [27,28]. In the acidic environment of endosomes, IgG is protected from degradation by lysosomal enzymes and is then transcytosed. This is followed by the fusion of endosomes with the syncytiotrophoblast membrane, cleavage of IgG from FcRn, and its release into the fetal circulation [29,30]. Maternal total IgG and specific antibody levels (very high concentrations of maternal IgG are limited in their transplacental transfer by a mechanism based on the saturation of the FcRn receptor), gestational age, placental status, IgG subclass (IgG1 and IgG4 are transported across the placenta more easily as they have the highest affinity for FcRn receptors), and antigen nature affect maternal IgG transfer across the placenta [26,31,32,33,34,35].

It has also recently been suggested that maternal IgE can also be transferred across the placenta mediated by FcRn in the form of IgG anti-IgE/IgE immune complexes [31,32].

Maternal immunization during pregnancy not only protects mothers but also fetuses by increasing transplacental transfer of maternal antibodies [33]. Moreover, although IgM does not cross the placental barrier, previous studies have reported the detection of tetanus toxoid-specific IgM in the umbilical cord blood of infants whose mothers were vaccinated during pregnancy [35,36]. Similarly, influenza vaccine-specific IgM has also been detected in umbilical cord blood after vaccination of pregnant women [37,38]. Such studies probably suggest that there is another way to protect the fetus from maternal antibody transfer, perhaps through sensitization of fetal B cells in response to maternal vaccination [33,38].

### 4.2. Cytokines

During pregnancy, any infection causes the production of cytokines such as IL-6 and TNF-α from the mother’s immune system [39]. The transfer of maternal inflammatory cytokines across the placenta secondarily induces the fetal response to maternal inflammation and T cell activation and differentiation, contributing to fetal immune development [40]. However, the overreaction of the fetus against inflammation may have the opposite effect and threaten the normal outcome of the pregnancy [41]. For example, a recent systematic search and meta-analysis have shown that inflammation and increased inflammatory biomarkers, such as the neutrophil-lymphocyte ratio during pregnancy, are significantly associated with gestational diabetes [42]. A correlation has also been found between inflammation and the development of maternal depression [43]. Furthermore, it has been suggested that cytokine disturbances during pregnancy may adversely affect fetal neurodevelopment [39,44].

### 4.3. Microchimeric Cells

#### 4.3.1. Microchimeric Cell Types

**Figure 1:**
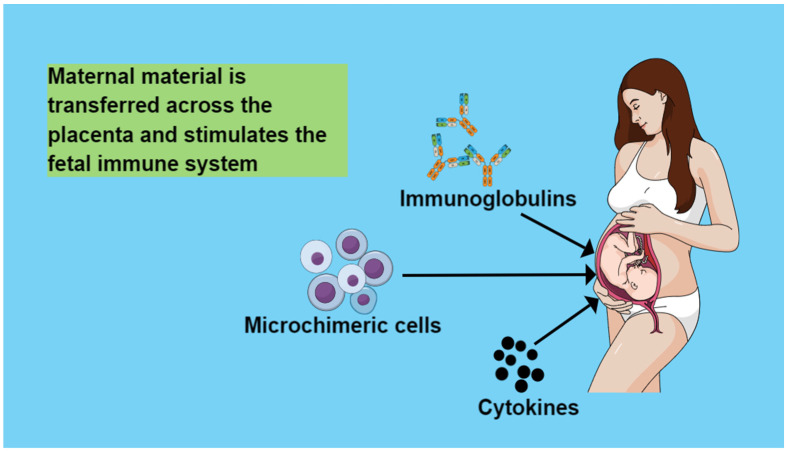
Maternal material stimulates fetal immune system.

During pregnancy, the placenta allows for the two-way exchange of both mature and progenitor cells between mother and fetus [45,46]. Thus, on the one hand, fetal cells are transferred to the mother’s circulation (fetal microchimerism FMc). On the other hand, maternal cells are transferred to the offspring (maternal microchimerism MMc), contributing in this way to the normal outcome of the pregnancy and preventing the immune conflict between mother and fetus [11]. Maternal cells are transferred to the fetus as early as the second trimester of pregnancy [45]. It has been found that genetically different fetal cells remain in maternal tissues for many years after pregnancy and that maternal cells persist in offspring tissues, even into adulthood [47,48] (Figure 1).

The cell types exchanged between mother and fetus include leucocytes, T cells, and progenitors of different lineages such as hematopoietic or mesenchymal stem cells or endothelial progenitors [48]. Kanold AMJ et al. showed that maternal cord blood microchimerism involved both lymphoid and hematopoietic progenitors, such as cellular subsets of CD3+ (T-cells), CD19+ (B-cells), CD33+ (myeloid cells), CD34+ (hematopoietic progenitor cells), and CD56+ (natural killer cells) cells [49,50]. They also detected MMc in total DNA [49]. Earlier studies had also identified MMc in CD3+, CD8+, memory, and progenitor T-cells, and in B-cells, NK cells, monocytes, and hematopoietic progenitor cells were detected [51,52].

#### 4.3.2. Factors Affecting Prevalence and Incidence of Maternal Microchimeric Cells

It is still unclear whether the mode of delivery and the umbilical cord clamping time play a role in the prevalence of MMc. Some studies found no differences between vaginal delivery and caesarean sections, while others indicated that the mode of delivery might affect MMc, with a higher prevalence in elective caesarean sections. [39,53,54]. It has also been argued that the duration of labor and labor contractions probably do not affect the incidence of MMc in the fetus, and that this MMc transfer occurs to a greater extent in the later stages of pregnancy [45,52]. It is not yet clear whether gender is associated with a higher frequency of MMc detection, but it seems likely that there are no gender differences [49,53,54].

Haddad ME et al. were the first to show that high levels of maternal serum PAPP-A (papalysin 1) concentrations in the first trimester were associated with an increased presence of MMc in umbilical cord blood. PAPP-A is a secreted metalloproteinase produced by the fetal syncytiotrophoblast cells, which promotes decidual vascularization. When the fetal syncytiotrophoblast cells secrete large amounts of PAPP-A, pressure is exerted on the maternal vessel resulting in increased fetal–maternal exchange and higher amounts of MMc in the cord blood [54].

#### 4.3.3. Maternal Microchimeric Cells between Generations

Berry et al. showed that MMc in the blood of second- and third-trimester fetuses was associated with maternal HLA-DRB1 and/or DQB1 compatibility from the child’s perspective [55]. Similarly, Haddad ME et al. showed that high levels of MMc in cord blood were associated with maternal HLA-DRB1, but also with HLA-A compatibility from the child’s perspective as well [54].

It is possible that MMc can be passed down through generations from the maternal grandmother. A study conducted by Gammill et al. suggests that interactions between a pregnant woman and her acquired microchimerism cell populations may influence normal reproductive processes. If these interactions become disrupted, they may contribute to or indicate immune dysfunction in preeclampsia [56]. Furthermore, since during pregnancy, there is an interaction and exchange of cells between mother and fetus, the mother who had received genetically foreign populations of fetal cells in a previous pregnancy can transfer them to subsequent pregnancies, and microchimeric cells from older siblings can be transferred to younger siblings. But even in utero, genetically different cells can be exchanged between dizygotic twins. Therefore, birth order may affect the amount and type of microchimeric cells that are passed down between generations [11,57].

#### 4.3.4. The Role of Maternal Microchimerism in Offspring’s Health

Maternal cells have also been detected in the offspring’s brain, although their effect there is not yet known. However, studies in mice have shown that maternal microchimerism probably regulates microglia homeostasis, prevents excessive presynaptic shedding, and contributes to the maturation of behavioral abilities [58].

Balle C et al. examined factors that may affect the detection and level of MMc during infancy and the effect of MMc on T cell responses to bacillus Calmette–Guérin (BCG) vaccination in a cohort of HIV-exposed, uninfected, and HIV-unexposed infants in South Africa. They found that MMc was present in most infants across infancy, and that it was positively impacted by the absence of maternal HIV, maternal and infant HLA compatibility, infant female sex, and exclusive breastfeeding. They also found that the level of MMc in uninfected HIV-exposed infants was partially restored by initiation of maternal antiretroviral therapy before pregnancy, and that MMc at birth was associated with an improved CD4+ T cell response to BCG, suggesting that MMc may influence infant T cell responses [59].

In addition, Castellan FS et al. hypothesized a possible postnatal regulation of lymphocytic responses by maternal microchimeric cells that could potentially confer protection to the neonate from future hypersensitivity reactions [60].

Stelzer IA et al. demonstrated that MMc may induce differentiation of hematopoietic stem cells in fetal bone marrow towards monocytes within the myeloid compartment, and that MMc leukocytes may promote neonatal immunity against early-life infections both in mice and humans [61].

Furthermore, immune MMcs appear to include pathogen-specific T cells from previous maternal infections and vaccinations, which could provide the neonate with pathogen-specific protection, even greater than maternal antibodies protection, due to the longevity of T cells [62,63]. Similarly, Yüzen D et al. showed that MMc may have the ability to transfer immunological memory between generations [64].

Moreover, Iwai S et al. demonstrated that mesenchymal stem cells/stromal cells (MSCs) as non-inherited maternal antigens (NIMAs) transferred by maternal microchimerism could induce immune tolerance and could serve as a basis for developing new therapies for autoimmune or genetic diseases [65].

#### 4.3.5. Microchimerism and Therapeutic Strategies

As mentioned above, maternal cells, particularly mesenchymal stem/stromal cells, play a key role in inducing immune tolerance to maternal antigens, thus preventing autoimmune responses and protecting fetal immune development. This feature of MSCs can potentially be exploited in new therapeutic strategies. Iwai S et al. showed in mouse models that immunization with MSCs could induce immune tolerance against MSC-specific antigens. Furthermore, they clarified that MMc-transferred MSCs could induce immune tolerance and that the degree of this immune tolerance could be enhanced by a drug [65]. In addition, Papait A et al. have also shown that placental MSCs have immune modulatory activity, and that the areas of the placenta from which they are isolated can influence the differentiation, angiogenesis, and the ability to inhibit the proliferation of T-lymphocytes, which is of particular importance in regenerative medicine and in the treatment of inflammatory and autoimmune disorders [66]. An example is expanded placental cells (PLX), which are specially designed for clinical application cells and supplied by Pluristem LTD, Israel. Both maternal (PLX-PAD) and fetal (PLX-R18) cells have been shown to have immunomodulatory properties. Between them, fetal-derived cells present a stronger ability to inhibit T-cell proliferation and cytotoxicity and induce the transition to M2 macrophages, while cells derived from the mother have a stronger ability to induce Tregs [66]. Their potential applications are also different: PLX-R18 embryonic cells have found application in a preclinical model of graft-versus-host disease (GVHD), while PLX-PAD cells, due to the high release of pro-angiogenic factors, vascular endothelial growth factor factor (VEGF) and angiopoietin have been tested in animal models of limb ischemia [67]. However, despite the good results of preclinical and clinical research studies, the treatment is still considered experimental [68]. Although it has been argued that fetal MSCs may have an advantage in therapeutic applications compared to maternally derived MSCs due to higher levels of human growth factor (HGF), both have been used in animal disease models with spectacular results [69]. For example, they have been shown to effectively inhibit the progression and metastasis of colon cancer, repair liver fibrosis, improve the systolic and diastolic function of the left ventricle in chronic heart failure, reduce the end-systolic diameter of the left ventricular mass, and increase the left ventricular ejection fraction in myocardial infarction. In addition, in such studies, MSCs were able to heal skin wounds, treat limb ischemia in diabetes through newly formed capillaries, dilated arterioles, and the secretion of various pro-angiogenic factors, cure experimental autoimmune encephalomyelitis by inhibiting T-cell proliferation and reducing IL-17 production, reduce the level of fibrosis in the diaphragm and cardiac muscles, and inhibit inflammation in Duchenne muscular dystrophy. It has also been hypothesized that MSCs may potentially reduce inflammation, increase immunomodulation, and promote the regeneration of damaged alveolar cells in patients with COVID-19 [70]. Moreover, they have a potential therapeutic role in cerebral palsy, autism, hypoxic-ischemic encephalopathy, spinal cord injuries, stroke, Type I and Type II diabetes, liver disease, congenital heart defects, multiple sclerosis, rheumatoid arthritis, ulcerative colitis, systemic lupus erythematosus, psoriasis, retinitis pigmentosa, and many others [71].

#### 4.3.6. Microchimerism and Future Perspectives

Despite the above encouraging results, further elucidation of the characteristics, the paracrine mechanisms of action, and their immunomodulatory properties of different MSCs is needed. In addition, the best culture conditions for their isolation and expansion under good manufacturing practices, the dose of cells to be used, and the treatment regimen for clinical trials should be determined. It would also be particularly beneficial to identify the molecules responsible for the therapeutic effects of MSCs and design a cell-free therapy without the potential risks that could arise from cell transplantation [71]. Since most human clinical trials of advanced perinatal cell therapies are at an early stage, future research is expected to achieve significant results. Biobanks are also expected to play an important role in the future in ensuring that perinatal cells reach the clinic safely while maintaining their efficacy. Nanotechnology could also help control the process of cell differentiation and proliferation, label and monitor transplanted cells, and facilitate drug delivery [72]. Furthermore, another challenge will be the development of nanocarriers for targeted drug delivery and on-site drug release that will improve efficacy and reduce side effects in adjacent tissues. Even genes and drugs could be incorporated into the nanoparticles, and these nanoparticles and perinatal MSC could be used as carriers to release the therapeutic complex whenever and wherever needed [73].

## 5. Maternal Microchimerism and Offspring’s Autoimmunity

Several studies have suggested that maternal microchimeric cells transferred during pregnancy may play a role in the offspring’s immunity later in life or even trigger the onset of an autoimmune disease.

### 5.1. Maternal Microchimerism and Type 1 Diabetes

Type 1 diabetes (T1D) is an autoimmune disease caused by the destruction of insulin-producing β-cells in the pancreas that appears to be influenced by many factors, both genetic and environmental, including viral, bacterial, and fungal infections, as well as early feeding, gut microbiome, and factors related to the mother [68,69,70].

It has been suggested that when there is a predisposition to T1D, environmental factors stress pancreatic β-cells, releasing β-cell antigens [74,75]. These antigens are processed by antigen-presenting cells (APCs) and presented via HLA Class II MHC molecules to naïve CD4+ T cells, which are activated and differentiated into Th1, releasing cytokines such as TNFα and INFγ into Th17, releasing inflammatory cytokines such as IL17 and IL-22 into immunosuppressive regulatory T cells (Tregs), which release anti-inflammatory IL-10 and TGFβ [76]. Th1 and Th17 activation also activates autoreactive cytotoxic CD8+ T cells, which migrate to the pancreas and secrete cytotoxic factors, such as granzyme B (GRZB), perforin (PRF), TNFα, and INFγ, resulting in the destruction of β-cells [77]. On the other hand, the release of the anti-inflammatory molecules IL-10 and TGFβ from Tregs blocks the autoreactive activity of CD8+ T cells, resulting in the inhibition of T1D [76]. The release of IFNγ from CD4 and CD8 cells may destroy β-cells and pancreatic islets while, together with other cytokines, it can further stimulate the secretion of chemokines from β-cells, increasing their vulnerability, and activate the death receptor FAS (known as CD95), triggering their apoptosis [78,79]. Moreover, the chemokines secreted by β-cells increase the recruitment of mononuclear cells to the site [76].

Nelson JL et al. had previously autopsied pancreases from young men with and without Type 1 diabetes and detected female (presumed maternal) cells capable of producing insulin. The authors hypothesized that these cells could potentially be the target of the immune response in Type 1 diabetes or an indicator of regeneration of damaged tissue. This study concluded that T1D patients had higher levels of MMc in their circulation than healthy siblings and other subjects, and that these cells could promote the generation of islet beta cells in the offspring [79].

Vanzyl B et al. further attempted to determine the levels of maternal microchimerism in pancreas samples from individuals with Type 1 diabetes compared to healthy controls and to phenotype these MMc using insulin as a marker of beta cells and CD45 as a marker of lymphocytes. In this study, both insulin-positive and insulin-negative MMc were detected, while no CD45-positive MMc were observed. Study results confirmed the presence of higher MMc levels in the T1D pancreas compared to healthy controls, assuming that maternal stem cells have the ability to cross the placental barrier and differentiate into both endocrine and exocrine cells without being immune effector cells [80].

Roy E et al. showed in mice that maternal chimeric T cells can have activity against the offspring pancreatic beta cells, causing islet inflammation that possibly predisposes to autoimmune diabetes [48].

Heninger AK et al. suggested that naïve T cells that can specifically respond to islet autoantigen are likely to be found among cord blood T cells since, as previously argued, autoreactive T cells develop in the thymus during fetal life, and low-affinity T autoreactive cells appear to be able to transfer to cord blood through negative selection [81].

Another study found that the degree of MMc in cord blood probably does not predict the risk of childhood-onset Type 1 diabetes [82].

**Figure 2:**
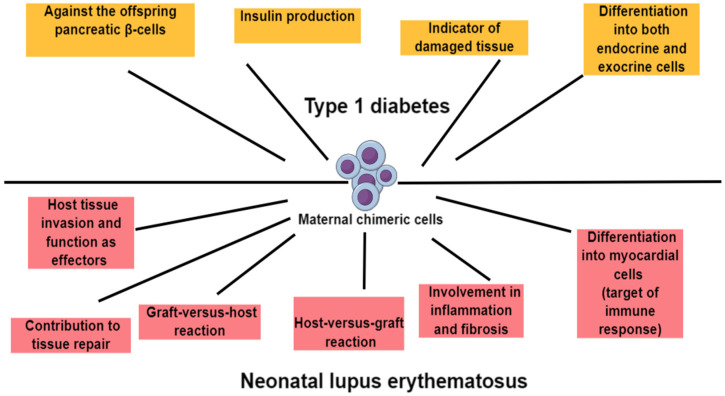
Maternal microchimerism is involved in offspring autoimmune diseases.

Ushijima K et al. suggested that there may be ethnic differences in the prevalence of maternal microchimerism in the peripheral blood of children with Type 1 diabetes, leading to different outcomes. They studied Japanese children with Type 1 diabetes, including β-cell autoantibody-positive children, and their healthy siblings with the aim of determining the prevalence and degree of maternal microchimerism. They found no differences in the prevalence or levels of maternal microchimerism between the study groups, while they found phenotypic similarities between those with and without maternal microchimerism. The study concluded that the low prevalence of maternal microchimerism in the peripheral blood of Japanese children with Type 1 diabetes suggests that it does not appear to play an important role in triggering the disease in this ethnic group in contrast to white Europeans, where it was previously found more frequently [83] (Figure 2).

### 5.2. Maternal Microchimerism and Juvenile Idiopathic Inflammatory Myopathies

Juvenile idiopathic inflammatory myopathies (JIIM) are a rare heterogeneous group of connective tissue diseases characterized by chronic muscle inflammation and progressive proximal and symmetric muscle weakness [84]. JIIM appears to develop when there is a genetic predisposition and after the influence of various environmental factors, affecting females up to two or three times more often than males. Between them, juvenile dermatomyositis (JDM) is the most common juvenile idiopathic inflammatory myopathy in childhood. The pathogenesis involves an attack of B lymphocytes, macrophages, and CD4+ T lymphocytes in the muscle capillaries, while polymyositis (PM), which is more common in adults, involves a large degree of focal muscle fiber destruction by CD8+ T lymphocytes [85,86]. JDM has been reported to share some features with chronic graft-versus-host disease (cGvHD), a condition due to incompatibility between the HLA genes from the donor and those of the recipient. Similarly, it has been hypothesized that JDM, which usually affects individuals carrying certain HLA genes, could be related to the reaction to HLA alleles from the invading microchimeric population [86,87].

Reed AM et al. investigated 15 children with juvenile dermatomyositis and their siblings to determine whether chimerism was associated with this disorder. Chimerism was confirmed by PCR in 13 of 15 children with the disorder and in five of 35 siblings. Maternal microchimeric cells among peripheral blood mononuclear cells were found in 11 of 15 boys with the disorder, in five of 17 controls, and in muscle tissue of 12 of 15 sufferers and two of ten controls. The authors argued that chimerism may thus be involved in juvenile dermatomyositis pathogenesis [87].

Yi Ye et al. also showed a higher frequency of MMc in muscles from children with juvenile dermatomyositis than in controls [88].

Artlett et al. had also previously shown that microchimeric cells were more frequent and more numerous in the peripheral blood and muscle tissue of patients with JIIM compared to non-inflammatory or control subjects, also suggesting that maternal microchimeric cells could be involved in the pathogenesis of JIIM [85,86]. Artlett CM et al. further demonstrated that maternal cells can still be detected in the peripheral blood of their offspring up to three decades after birth and that subjects’ ethnicity and gender, as well as prednisone treatment, do not appear to affect microchimerism status [85]. The authors noted that the prevalence and extent of maternal microchimerism in the peripheral blood of healthy subjects was found to be much lower than the fetal microchimerism found in earlier studies in the peripheral blood of healthy multiparous women. It was argued that these differences may be related to the research method that may lead to false negative results, or that children can acquire chimeric cells only once in utero, compared to mothers, who can acquire chimeric cells both in utero and during their pregnancies, or even that fewer maternal microchimeric cells are possibly transferred to the fetus across the placenta than vice versa [85]. It was also hypothesized that specific environmental factors could affect both the immune system and the frequency of detection of microchimeric cells, the duration of which was also related to time since these cells multiply at a slow rate. Additionally, it was suggested that immunosuppressive treatments may reduce the number of microchimeric cells below the detection limit. It was also hypothesized that the number of maternal chimeric cells may be greater at birth and decline thereafter maybe because of their damage to the newborn or because of their short life span. They may also not be detected in the peripheral circulation due to their attachment in the bone marrow, spleen, and liver [85].

In a more recent study, Artlett et al. further investigated microchimeric cells in JIIM, compared to muscular dystrophy (MD), which is an inflammatory but non-autoimmune muscle disease, and to non-inflammatory control muscles, and examined the immunophenotypes of microchimeric cells in muscle tissue. It is interesting that they came to opposite results with their previous studies, since they showed that microchimeric cells probably do not participate in the pathogenesis of JIIM, which is an autoimmune disorder, but neither of MD, which is a non-autoimmune inflammatory disease. They also showed that the microchimeric cells are probably not involved in the repair of the muscle tissue, but rather they, together with the autologous cells, after their differentiation, are recruited to the sites of inflammation in a similar way [89].

### 5.3. Maternal Microchimerism and Systemic Lupus Erythematosus

During pregnancy, maternal anti-Sjögren’s-syndrome-related antigen A (anti-SSA/Ro), anti-Sjögren’s-syndrome-related antigen B (anti-SSB/La), or anti-U1 ribonucleoprotein (anti-U1-RNP) antinuclear autoantibodies can cross the placenta and cause the newborn an autoimmune disorder called neonatal lupus erythematosus (NLS). The main clinical symptoms are skin lesions, congenital heart block (CHB), biliary liver damage, thrombocytopenia, and neutropenia. Apart from these, for the correct diagnosis of the disease, the presence of antibodies in the serum of the mother or the infant is also required. It is characteristic that the symptoms, apart from the congenital heart block, may disappear after the clearance of maternal antibodies [90].

A previous study demonstrated a myocardial tissue-specific maternal microchimerism phenotype in neonatal lupus congenital heart block [91].

Stevens AM et al. suggested that maternal autoantibodies are probably not the exclusive causative factors for the development of NLS, and that maternal microchimeric cells could also play an important role, since on the one hand, most pregnant women with anti-SSA antibodies and/or SSB give birth to apparently healthy infants, and on the other twins, both exposed to the same maternal autoantibodies, do not show the same outcome [92]. The authors also argued that maternal microchimeric cells are probably found much more frequently in the tissues and mainly in the myocardium than in the blood of CHB neonates [91]. They suggested that maternal microchimerism could be involved in inflammation and fibrosis, and, in combination with autoantibodies and other factors, they could contribute to the pathogenesis of NLS [93]. The authors assumed that the maternal cells, which had differentiated into myocardial cells, could act within tissues as immune targets contributing to disease pathogenesis. Allogeneic somatic cells could also provide HLA proteins and other allelic proteins, which could promote a chronic inflammatory response. Moreover, maternal blood cells could also invade host tissues and function as effectors, or maternal cells could participate in tissue repair [92].

Similarly, I. C. L. Kremer Hovinga et al. proposed three hypotheses regarding the role of chimeric cells in autoimmunity and the pathogenesis of SLE. The first hypothesis is that the chimeric cell is a T cell that induces a graft-versus-host (GVH) reaction. According to this scenario, there is no cytotoxic T-cell (CTL) reaction of chimeric Tc cells (cytotoxic T cells) against SLE-specific antibodies producing B cells nor against SLE-specific antigens, with consequences of the continuous stimulation and proliferation of the host B cells by the chimeric Th (T helper) cells, and, hence, the uncontrolled production of the specific antibodies [94].

The second hypothesis is that the chimeric cell induces a host-versus-graft (HVG)-like reaction. This can occur directly, when a chimeric antigen is presented via host APCs to a host Th cell, causing activation of complement, host Tc cells, and host B cells, which either can lead to limited inflammation and disease, e.g., glomerulonephritis, in case of successful immune response locally, or in chronic inflammation and disease in case of insufficient response of the immune system and uncontrolled stimulation of B cells [94]. Chimerism can also indirectly induce an HVG reaction. A chimeric antigen is presented via a host APC to a host Th cell. However, if this foreign antigen shares epitopes with the recipient’s self-antigens, Th cells can be activated for these self-antigens and stimulate B cells to produce autoantibodies. Molecular mimicry leads to cross-reactivity of chimeric antigens and self-antigens, which causes the development of autoimmunity.

In the third hypothesis, the chimeric cell is not directly involved in the pathogenesis of the autoimmune disease, but it seems to contribute to tissue repair. This is probably achieved because the chimeric cells develop from progenitor cells into parenchymal cells and take the place of the injured tissue cells of the host. However, it has been hypothesized that after the first period of successful response to tissue damage, there is the possibility to initiate an HVG-type immune response at a later stage [95,96] (Figure 2).

### 5.4. Maternal Microchimerism and Sjogren’s Syndrome

Kuroki M et al. demonstrated for the first time that maternal-fetal microchimerism was found in the salivary glands and affected the lungs of female patients with Sjögren’s syndrome. Furthermore, they hypothesized that non-host cells may have played a role in the pathogenesis of Sjögren’s syndrome, as they were only present in the inflammatory lesions but not in peripheral blood [96]. Although in many autoimmune diseases, such as progressive systemic sclerosis, Hashimoto’s thyroiditis, Systemic lupus erythematosus, and Sjögren’s syndrome, fetal Y chromosomes have been detected decades after pregnancy. Insufficient evidence has yet been found for the involvement of maternal microchimeric cells in offspring autoimmunity [1].

Another study identified maternal chimersm in 72% of patients with systemic sclerosis and 22% of controls [97]. It has also been shown that maternal chimeric effector T cells and lymphocyte reactions between patients and their mothers may play a role in the pathogenesis of biliary atresia [98]. Moreover, in another study, rheumatoid nodules of rheumatoid arthritis patients were found to contain microchimerism with evidence of a fetal or maternal source, suggesting an association of microchimerism with the pathogenesis of this autoimmune disease [99]. In contrast, MMc tested in cord blood at birth was not found to be associated with a later risk of developing celiac disease in childhood [100].

## 6. Future Research

Further elucidation of the characteristics, mechanisms of action, and immunomodulatory properties of maternal microchimeric cells is essential. Future studies should prioritize rigorous experimental design and advanced technical approaches to ensure accurate detection and quantification of these cells. Additionally, research should explore potential therapeutic benefits of maternal microchimeric cells, such as their role in insulin production for diabetic offspring or their contributions to tissue repair and regeneration. Due to the multifactorial nature of autoimmune diseases, future investigations must also incorporate a broader range of variables, including genetic predispositions, environmental factors, and other unmeasured influences, to provide a more comprehensive understanding of how maternal microchimerism interacts with these elements in disease development and progression.

## 7. Strengths and Limitations

This review provides a comprehensive analysis of the current evidence supporting the association between maternal microchimerism and the development of autoimmune diseases. It also emphasizes the consistency of results across different populations and study designs, which strengthens the case for maternal microchimerism as a potential contributor to autoimmunity. The review successfully identifies key areas where the evidence is robust, particularly in the context of specific autoimmune conditions, and integrates recent findings to present a clear overview of the current understanding in this field. However, this review also acknowledges significant limitations in the existing body of research. The primary limitation of studies investigating the role of maternal microchimerism in autoimmune disease development lies in the challenges associated with detecting and quantifying microchimeric cells, due to their low frequency and presence in healthy individuals. Variability in assay sensitivity and specificity across different studies can lead to inconsistent results, making it difficult to draw definitive conclusions. Additionally, the small sample sizes, lack of standardized methodologies, and differences in study design, such as the timing of sample collection and selection of controls, further complicate the interpretation of findings. These limitations highlight the need for more rigorous, standardized research to better understand the significance of maternal microchimerism in autoimmunity. While the review provides a valuable synthesis of current knowledge, it also underscores the need for continued investigation to address these gaps and refine our understanding of this complex phenomenon.

## 8. Conclusions

Much of the literature focuses on the impact of fetal microchimerism on maternal health, with fewer studies investigating the effects of maternal microchimerism on offspring health. Opinions on this matter are divided. As previously discussed, maternal microchimeric cells may potentially contribute to autoimmune diseases such as Type 1 diabetes and systemic lupus erythematosus. According to our findings, maternal microchimeric cells have been related to Type 1 diabetes either acting against the offspring pancreatic β-cell, producing insulin, differentiating into endocrine and exocrine cells, or simply being indicators of tissue damage. In addition, maternal cells have been linked to neonatal lupus erythematosus, either causing in the offspring a graft-versus-host or host-versus-graft reaction, acting in the tissues as effectors, or contributing to tissue healing. They have also been found to be involved in the process of inflammation and fibrosis and to differentiate into myocardial cells triggering the immune response. In addition, maternal microchimeric cells may potentially contribute to juvenile idiopathic inflammatory myopathies, Sjögren’s syndrome, systemic sclerosis, biliary atresia, and rheumatoid arthritis. Conversely, no association of maternal cells with offspring celiac disease has been found.

Therefore, the exact role of MMc in these conditions remains unclear, necessitating further research to elucidate its mechanisms and implications.

## Data Availability

The original contributions presented in the study are included in the article, further inquiries can be directed to the corresponding authors.
